# Delay in diagnosis of cancer as a patient safety issue - a root cause analysis based on a representative case report

**DOI:** 10.1186/1754-9493-5-19

**Published:** 2011-07-29

**Authors:** Subramanian Vaidyanathan, Bakul M Soni, Gurpreet Singh, Peter L Hughes, Paul Mansour, Tun Oo

**Affiliations:** 1 Regional Spinal Injuries Centre, Town Lane, Southport, Merseyside PR8 6PN, UK; 2 Department of Urology, District General Hospital, Town Lane, Southport, Merseyside PR8 6PN, UK; 3 Department of Radiology, District General Hospital, Town Lane, Southport, Merseyside PR8 6PN, UK; 4 Department of Cellular Pathology, District General Hospital, Town Lane, Southport, Merseyside PR8 6PN, UK

**Keywords:** Spinal cord injury, Urinary bladder, Carcinoma, Suprapubic cystostomy, Cystoscopy

## Abstract

**Background:**

It is well known in the literature that imaging has almost no value for diagnosis of superficial bladder cancer. However, wide gap exists between knowledge on diagnosis of bladder cancer and actual clinical practice.

**Case presentation:**

Delay in diagnosis of bladder cancer in a male person with tetraplegia occurred because of reliance on negative flexible cystoscopy and single biopsy, negative ultrasound examination of urinary bladder, and computerised tomography of pelvis. Difficulties in scheduling cystoscopy also contributed to a delay of nearly ten months between the onset of haematuria and establishing a histological diagnosis of vesical malignancy in this patient. The time interval between transurethral resection and cystectomy was 42 days. This delay was mainly due to scheduling of surgery.

**Conclusion:**

We learn from this case that doctors should be aware of the limitations of negative flexible cystoscopy and single biopsy, cytology of urine, ultrasound examination of urinary bladder, and computed tomography of pelvis for diagnosis of bladder cancer in spinal cord injury patients. Random bladder biopsies must be considered under general anaesthesia when there is high suspicion of bladder cancer. Spinal cord injury patients with lesions above T-6 may develop autonomic dysreflexia; therefore, one should be extremely well prepared to prevent or manage autonomic dysreflexia when performing cystoscopy and bladder biopsy. Spinal cord injury patients, who pass blood in urine, should be accorded top priority in scheduling of investigations and surgical procedures.

## Background

Persons with spinal cord injury, who have been managing neuropathic bladder with indwelling urinary catheter for several years, are at risk for developing vesical malignancy. It is well known in the literature that imaging has almost no value for diagnosis of superficial bladder cancer. Flexible cystoscopy and biopsy are carried out to detect bladder neoplasm in spinal cord injury patients. But flexible cystoscopy and biopsy may prove to be a sub-optimal procedure in some spinal cord injury patients.

• In persons with false passage in urethra, insertion of flexible cystoscope through urethra may be difficult or even impossible. When flexible cystoscopy is performed through suprapubic cystostomy, the region of the bladder immediately below suprapubic cystostomy may escape the attention of endoscopist unless the urologist makes special efforts to visualise this site.

• Patients with long-term indwelling catheters may have lot of sediments in urine; debris and sediments may obscure the vision during cystoscopy.

• Mucosa of neuropathic bladder may be oedematous and bleed easily during flexible cystoscopy when the bladder is distended with irrigating fluid, thus obscuring vision.

• Very rarely, the flexible cystoscope may be defective; fibre optic cables may be broken, or the tip of cystoscope may not bend as it should.

• When biopsy of bladder mucosa is taken with flexible cystoscopy, no stroma may be included in the tiny specimen, thus the specimen may be unsuitable for assessment of possible low grade neoplasia.

We describe a spinal cord injury patient in whom reliance on (1) sub-optimal cystoscopy and single biopsy, (2) negative ultrasound imaging and computed tomography of pelvis, led to considerable delay in diagnosis of bladder cancer. This case illustrates the wide gap which exists between knowledge on diagnosis of bladder cancer and actual clinical practice that is feasible in day-to-day set-up.

## Case Presentation

In May 1975, a 22-year-old Asian male person was helping to lift a beam with his co-workers, when the whole load of the beam and bar together struck against his left shoulder. He felt severe pain locally and developed complete tetraplegia at C-4 level. This person had been managing neuropathic bladder by indwelling urethral catheter size 12 French. Over a period of time, he developed erosion of urethra because of indwelling urethral catheter. Therefore, suprapubic cystostomy was performed in November 2008. Cystoscopy showed trabeculated bladder and no tumour was visible.

This person was never a smoker and was not a second-hand smoker. On 28 December 2009, this person started passing blood in urine. Ultrasound examination of urinary tract, performed on 29 December 2009, revealed right kidney measuring 10.3 cm and left kidney measuring 9.6 cm. There was mild right hydronephrosis and slight dilatation of the left collecting system. No renal calculi were seen. The outline of urinary bladder was normal with suprapubic catheter in situ.

**Figure 1:**
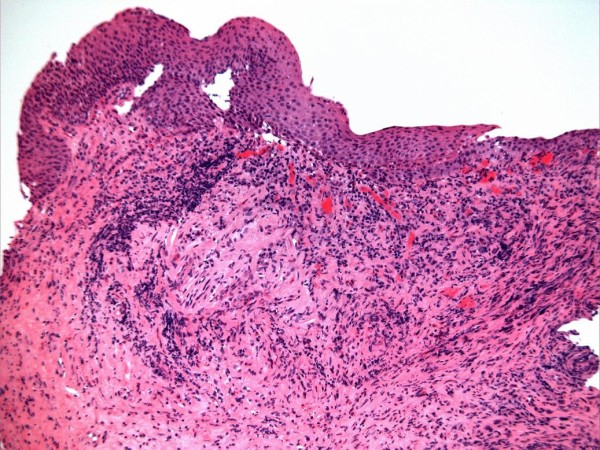
**Bladder biopsy taken with flexible cystoscope**. Bladder biopsy (HP10/04115) from April 2010 showing non-keratinising squamous metaplasia of surface epithelium (top) with non-specific stromal inflammation; there is no evidence of malignancy. (H&E stain)

In April 2009, flexible cystoscopy was performed through suprapubic cystostomy track, as false passages in urethra prevented insertion of cystoscope through urethra. Reddened areas were noted in the dome and two biopsies were taken. Histological sections were examined at multiple levels. Mild, active, chronic inflammatory cell infiltrate was seen (Figure 1). A fragment in part showed squamous metaplasia of surface epithelium. Towards one edge, surface urothelium showed mild nuclear enlargement and no staining for cytokeratin 20 was observed. This most likely represented inflammatory or reactive atypia rather than genuine dysplasia. There was no evidence of carcinoma-in-situ.

**Figure 2:**
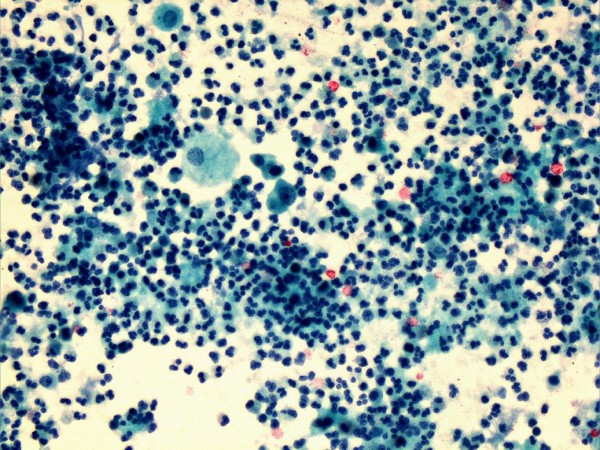
**Urine cytology**. Urine cytology (Papanicolau stain) from July 2010 (NG10/00706) showing large numbers of neutrophil polymorphs together with scattered urothelial cells (centre & top left), which show mild atypia only.

This patient continued to pass blood in urine. Therefore, a sample of urine was sent for cytology. Cytology revealed a very cellular specimen containing numerous neutrophil polymorphs together with scattered red blood cells. The transitional epithelial cells in this specimen showed mild nuclear enlargement, although the nuclear cytoplasmic ratio was not altered. In addition, scattered abnormal shaped squamous cells were present showing orangiophilic cytoplasm, containing mildly pleomorphic nuclei. These epithelial/nuclear changes were probably inflammatory, metaplastic and reactive in nature (Figure 2). However, the atypia in these cells warranted further investigations, for example cystoscopy.

**Figure 3:**
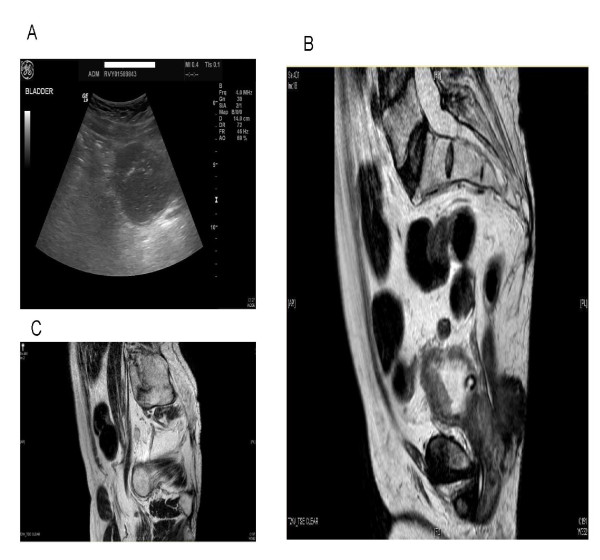
**Ultrasound scan and MRI of pelvis**. **A **Ultrasound scan of urinary bladder (23 July 2010): 150mls sterile saline instilled into the urinary bladder via suprapubic catheter: No gross bladder abnormality was demonstrated. Small amount of debris noted within the bladder. **B **MRI of pelvis performed on 29 October 2010 shows 24 mm thick anterior bladder wall tumour involving the full thickness of the bladder wall. There is streaking of the peri vesical fat anteriorly consistent with extravesical extension. **C **MRI of pelvis performed on 29 October 2010 showed 15 mm × 10 mm left iliac lymph node. No other pelvic lymphadenopathy was seen.

In July 2010, ultrasound examination of urinary bladder was performed after instilling 150 ml of sterile 0.9% sodium chloride solution into the urinary bladder through suprapubic catheter. No gross bladder abnormality was demonstrated. Small amount of debris was noted within the urinary bladder. (Figure 3-A) Repeat ultrasound examination, performed in August 2010, revealed normal position of the kidneys, no hydronephrosis or focal renal abnormality. The urinary bladder was empty.

Flexible cystoscopy was performed after four months after the previous cystoscopy. Flexible cystoscope was again introduced through suprapubic cystostomy track. Vision was poor, as the flexible cystoscope was damaged. A biopsy was taken from sloughing area. On this occasion, flexible cystoscopy was considered to be a sub-optimal procedure. Histological sections showed a piece of fibrino-purulent exudates, presumably from the surface of an ulcer, intermingled with superficial fragments of urothelium, which showed inflammatory changes only. There was no evidence of high grade neoplasia or carcinoma-in-situ. One or two of the fragments had a possibly papillary pattern, raising the possibility of a low grade papillary urothelial tumour, but virtually no stroma was included and the specimen was unsuitable for assessment of possible low grade neoplasia.

A specimen of urine was sent in cytospin fixative. Cytology of urine specimen showed essentially blood and inflammatory cells only, with only one or two epithelial cells included. The specimen was therefore unsuitable for assessment.

Computed Tomography of abdomen and pelvis, performed on 14 September 2010, revealed small simple cortical cyst in left kidney and a small opaque calculus in lower pole of right kidney. There was mild dilatation of right renal pelvis and proximal ureter. There was no opaque ureteric calculus. Urinary catheter was in situ. There was no pelvic pathology.

This patient continued to pass blood in urine. Therefore, a sample of urine was sent for cytology on 14 October 2010, which revealed singly lying and some monolayered clusters of transitional epithelial cells, in which the nuclei were showing a mild degree of pleomorphism, many with prominent nucleoli. Large numbers of acute inflammatory cells were present in the background, suggesting that the nuclear changes seen in the transitional epithelial cells were likely reactive/inflammation induced. However, the nuclear atypia was significant in many cells and, therefore, it was advised to follow this patient and repeat the urine examination at appropriate interval.

In view of cytological findings of significant nuclear atypia in many transitional epithelial cells, rigid cystoscopy was performed on 22 October 2010. Cystoscope was introduced with difficulty, as false passages were present in prostatic urethra. A growth was seen in the anterior wall of urinary bladder under the suprapubic cystostomy. Transurethral resection was performed. Histology showed multiple pieces of a poorly differentiated transitional cell carcinoma with a solid growth pattern. Large areas of necrosis were noted. There was invasion of lamina propria but no definite muscle was identified. There was no carcinoma-in-situ.

Magnetic resonance imaging of urinary bladder, performed on 29 October 2010, revealed 24 mm thick anterior bladder wall tumour involving full thickness of the bladder wall (Figure 3-B). There was streaking of the perivesical fat anteriorly, consistent with extravesical extension. 15 mm × 10 mm left iliac lymph node was noted. (Figure 3-C) No other pelvic lymphadenopathy was present.

Computed tomography of chest, performed on 27 October 2010, showed clear lungs and no pulmonary metastases. Isotope bone scan revealed some focal increased tracer uptake in the right second rib posteriorly, which was of uncertain significance. There was increased uptake at the left gleno-humeral joint, which was probably related to an arthropathy.

**Figure 4:**
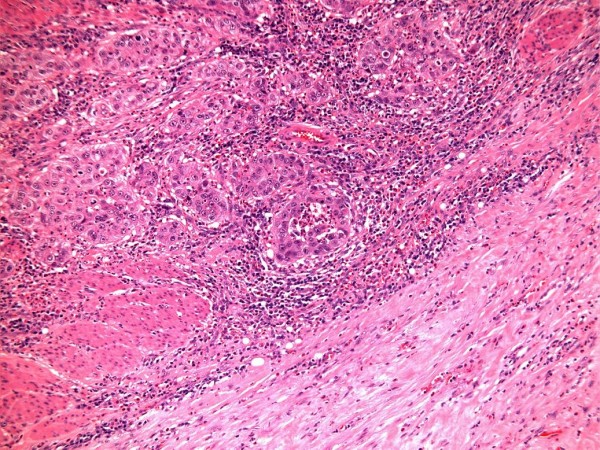
**Histology of radical cystectomy**. Section from radical cystectomy (HP10-14847) H&E stain showing grade 3 urothelial carcinoma (top and centre) infiltrating through bundles of muscularis propria (bottom left)

This patient underwent radical cystectomy on 03 December 2010, nearly eight months after initial flexible cystoscopy. Sections of urinary bladder confirmed a poorly differentiated, grade 3 solid urothelial carcinoma at the fundus of the bladder, which extended just through the muscularis propria into perivesical fat but without causing an obvious extravesical mass (Figure 4). Perineural invasion was present. The surface urothelium showed limited carcinoma in situ.

Post-operative period was complicated by sepsis and abdominal distension. Twelve days after cystectomy, computed tomography of abdomen revealed markedly distended small bowel loops; small bowel dilatation was up to 5 cm. There was significant fluid collection on the right side of the abdomen: 15 cm in craniocaudal extent, 6.5 cm in transverse diameter and 5.5 cm in antero-posterior diameter. There was a small right subphrenic fluid collection and a 5 cm deep fluid collection in the pelvis. Whilst the right kidney previously had a prominent extra renal pelvis this had increased in prominence since the previous computed tomography examination of September 2010 and there was now right calyceal dilatation. The patient also developed moderate left hydronephrosis. Both ureters could be traced down to their insertion into the ileal conduit. Clinical history and computed tomography appearances suggested an anastomotic leak. The collection in pelvis was not accessible for percutaneous drainage because of overlying bowel loops. This patient was managed conservatively and he improved. Five weeks after cystectomy, ultrasound examination of abdomen revealed no hydronephrosis or perirenal collection.

## Discussion

In this patient, reliance on negative flexible cystoscopy and biopsy, ultrasound examination of urinary bladder, and computed tomography of pelvis led to a delay in diagnosis of bladder cancer. It is well known in the literature that imaging has almost no value for diagnosis of superficial bladder cancer. Only in cases with high stage disease, will imaging studies demonstrate lymphadenopathy and/or invasive disease. A delay in scheduling of cystoscopy also contributed to delay in this patient. Flexible cystoscopy was a sub-optimal procedure in this case due to defective equipment. Random bladder biopsies must be performed under general anaesthesia when there is high suspicion of bladder cancer. In this patient, only a single biopsy was taken under no anaesthesia.

A delay in the diagnosis of bladder cancer has been shown to increase the risk of death from disease independent of tumour grade and or disease stage. Hollenbeck and associates [1] used the Surveillance, Epidemiology, and End Results-Medicare linked database for the years 1992 through 2002 to identify 29,740 patients who had haematuria in the year before a bladder cancer diagnosis and grouped them according to the interval between their first claim for haematuria and their bladder cancer diagnosis. Patients (n = 2084) who had a delay of 9 months were more likely to die from bladder cancer compared with patients who were diagnosed within 3 months (adjusted hazard ratio: 1.34; 95% confidence interval, 1.20-1.50).

In our patient, histological diagnosis of vesical malignancy was achieved nearly ten months after the first onset of haematuria (28 December 2009 to 22 October 2010). This delay could have been reduced considerably had we not relied on negative biopsy on flexible cystoscopy, and negative ultrasound scan or computed tomography of urinary bladder when this patient continued to pass blood in urine. Further, there was delay in scheduling cystoscopy and biopsy, and subsequently, cystectomy for this patient.

Before undergoing radical cystectomy and urinary diversion, which is a major surgical procedure, spinal cord injury patients often require extensive medical evaluation and clearance, and significant preoperative counseling. Beyond resolution of clinical barriers, spinal cord injury patients must achieve mental acceptance of the drastic change in voiding and potential affect on their body image conferred by a radical cystectomy and urinary diversion. This delay between diagnosis of muscle invasion and cystectomy has been shown to have serious consequences [2]. Studies suggested a window of opportunity of less than 12 weeks from diagnosis of invasive disease to radical cystectomy [3] Delay in definitive surgical treatment beyond 12 weeks conferred an increased risk of disease-specific and all-cause mortality among subjects with stage II muscle-invasive bladder cancer. A cystectomy delay of 3.1 months undermines patient survival, likely through the development of micrometastases, since local stage progression is not apparent at this point. Most delays are avoidable and should be minimized. Despite the need for second opinions and the impact of busy surgical schedules clinicians must strive to schedule patients efficiently and complete surgical treatment within this time frame [4]

Recently, Kulkarni and colleagues [5] analysed data from 2,535 patients who underwent radical cystectomy for bladder cancer during a 12-year period. The median wait time between transurethral bladder resection and the cystectomy was 50 days. The results suggested that 40 days was the ideal maximum wait time. Longer wait times were associated with a poorer overall survival rate. Patients, who had a cystectomy delay of more than 93 days faced about twice the risk of patients who had an earlier cystectomy, of dying from any cause or from bladder cancer, according to a multivariate analysis. Nearly half the scheduling delays in patients who underwent cystectomy after 93 days were related to clinical or research appointments. Patient co-morbidities accounted for 15 percent of the delays, and difficulty with decision-making contributed to 12 percent of the delays [4]. In our patient, the time interval between transurethral resection and cystectomy was 42 days (22 October to 03 December 2010). This delay was mainly due to scheduling of surgery. In order to minimise the delay from initial diagnosis to radical cystectomy, timely metastatic evaluations and immediate, yet thorough, medical evaluations before surgery should be carried out. Surgical schedules should be modified in order to minimise delay in performing cystectomy in a spinal cord injury patient with bladder cancer. This requires a concerted effort by medical managers and all health professionals.

We performed urine cytology in this patient to detect bladder cancer; however, cytology of catheterised urine sample in a person with spinal cord injury has low sensitivity. Several soluble and cell-based markers have been developed and most of them improve the sensitivity of cytology but the specificity is invariably decreased. Two cell-based tests have been approved by Food and Drug Administration of USA; ImmunoCyt/uCyt is a fluorescent test that uses three monoclonal antibodies and UroVysion is an in situ hybridization test, which uses four different probes to different chromosomes. Both tests have a high sensitivity to detect cancer cells [6].

Bravaccini and associates [7] assessed the diagnostic performance of (i) telomerase activity detected by telomeric repeat amplification protocol (TRAP) assay, (ii) cytology and TRAP assay in parallel, (iii) cytology in parallel with the in-series combination of TRAP assay and fluorescence in situ hybridization assay (FISH) analysis, and (iv) the in-series combination of TRAP assay and FISH analysis. 289 consecutive patients, who presented with urinary symptoms at a north Italian hospital between 2007 and 2008, underwent cystoscopy and cytology evaluation, and conclusive results were available for TRAP assay and FISH analysis. Compared with cytology alone, the combination of cytology, TRAP, and FISH provided the best trade-off between increase in sensitivity and loss in specificity. We did not have facilities to use any of these tests.

We performed ultrasound examination of urinary bladder after filling the bladder with 150 ml of sterile 0.9% sodium chloride solution; no gross bladder abnormality was seen. Nicolau and colleagues [8] found contrast-enhanced ultrasound to provide higher accuracy than ultrasound scan for bladder cancer detection, being especially useful in non-conclusive ultrasound studies.

## Conclusion

This case illustrates the wide gap, which exists between knowledge on diagnosis of bladder cancer and actual clinical practice that is feasible in day-to-day set-up. We learn from this case that doctors should be aware of the severe limitations of (1) sub-optimal flexible cystoscopy and single biopsy, (2) negative cytology of urine, (3) negative ultrasound examination of urinary bladder, and computed tomography of pelvis for diagnosis of bladder cancer in spinal cord injury patients. If a spinal cord injury patient continues to pass blood in urine, a senior urologist should perform cystoscopy under anaesthesia and take *multiple *biopsies. The doctor should be extremely well prepared to prevent or manage autonomic dysreflexia when performing cystoscopy in spinal cord injury patients with lesion above T-6. Further, spinal cord injury patients, who pass blood in urine, should be accorded top priority in scheduling of investigations and surgical procedures.

## Consent

Written informed consent was obtained from the patient for publication of this Case report and any accompanying images. A copy of the written consent is available for review by the Editor-in-Chief of this journal.

## Competing interests

The authors declare that they have no competing interests.

## Authors' contributions

SV conceived the idea and wrote the manuscript. GS performed cystectomy. PH reported medical images. PM reported histology of bladder biopsies, urine cytology and cystectomy specimen. BMS was the consultant in charge of the patient. TO participated in care of this patient. All authors read and approved the final manuscript.
